# Photopatternable Epoxy-Based Thermosets

**DOI:** 10.3390/ma12152350

**Published:** 2019-07-24

**Authors:** Michael Giebler, Simone Radl, Thomas Ules, Thomas Griesser, Sandra Schlögl

**Affiliations:** 1 Polymer Competence Center Leoben GmbH, Roseggerstrasse 12, A-8700 Leoben, Austria; 2 Institute of Chemistry of Polymeric Materials, Montanuniversitaet Leoben, Otto Glöckel-Strasse 2, A-8700 Leoben, Austria

**Keywords:** photopatterning, epoxy-based thermosets, photocleavage, *o*-nitrobenzyl ester, surface hardness, positive tone photoresists

## Abstract

The present work provides a comparative study on the photopatterning of epoxy-based thermosets as a function of network structure and network mobility. Local switching of solubility properties by light of a defined wavelength is achieved by exploiting versatile *o*-nitrobenzyl ester (*o*-NBE) chemistry. *o*-NBE derivatives with terminal epoxy groups are synthetized and thermally cured with different types of cycloaliphatic anhydrides via nucleophilic ring opening reaction. By varying the structure of the anhydride, glass transition temperature (*T*_g_) and surface hardness are adjusted over a broad range. Once the network has been formed, the photolysis of the *o*-NBE groups enables a well-defined degradation of the 3D network. Fourier transform infrared (FT-IR) spectroscopy studies demonstrate that cleavage rate and cleavage yield increase with rising mobility of the network, which is either facilitated by inherent network properties (*T*_g_ below room temperature) or a simultaneous heating of the thermosets above their *T*_g_. The formation of soluble species is evidenced by sol-gel analysis, revealing that low-*T_g_* networks are prone to secondary photoreactions at higher exposure doses, which lead to a re-crosslinking of the cleaved polymer chains. The change in solubility properties is exploited to inscribe positive tone micropatterns within the thermosets by photolithographic techniques. Contrast curves show that the resist performance of rigid networks is superior to flexible ones, with a contrast of 1.17 and a resolution of 8 µm.

## 1. Introduction

Due to their permanent network structure, classic epoxy-based thermosets are characterized by superior mechanical properties, high resistance to creep, and high thermal stability. These salient features make them ideal candidates for structural applications [1] and indispensable materials in functional coatings [2], electrical [3], and electronic devices [4]. The network formation typically relies on thermal curing routes with amines, amides, anhydrides, phenols, thiols, or imidazoles as crosslinkers [5]. The material properties of epoxy-based thermosets are governed by the structure, the curing parameters and the stoichiometric ratio both of monomer and crosslinker [6]. The curing reaction is often accelerated by the addition of selected organic molecules such as metal acetyl acetonates and tertiary amines [7]. 

Advancing from epoxy-based thermosets with permanent covalent crosslinks, recent research is geared towards the introduction of exchangeable chemical bonds and dynamic crosslinks, which offers a promising strategy for reshaping [8], self-healing [9,10], reprocessing [11,12], or recycling [13] of thermosets. 

Besides dynamic crosslinks, a convenient switching of network properties is realized by introducing irreversibly cleavable chromophores. Benefiting from a spatially controlled cleavage of covalent links by light exposure, the photoisomerization of *o*-NB alcohol derivatives has become a prominent strategy to impart photo-responsive properties to polymeric structures. Along with the preparation of photosensitive hydrogels [14], copolymers [15,16,17,18], or self-assembled monolayers [19], the application of *o*-NBE chemistry is of particular interest in the fabrication of patterned polymer structures and resist technology.

Various routes toward the synthesis of functional polymers for photoresists have been described, which employ *o*-NB alcohol derivatives as photocleavable binding motif. Successful approaches involved the introduction of *o*-NB ethers in the polymer backbone of poly(acrylates) and poly(ethylene glycol)s or the synthesis of poly(imide) precursors with pendant *o*-NB groups [20,21]. In addition, Ober and co-workers demonstrated the synthesis of a copolymer with perfluorinated methacrylate and *o*-nitrobenzyl methacrylate units, in which patterns in the sub-micrometer range were inscribed due to the ultra-violet (UV) light induced change in solubility [22]. The implementation of *o*-NB-based photoresists in organic electronics was also shown by Zojer and co-workers, who applied poly(norbornene)s with pendant *o*-NBE groups as photoreactive layers in organic thin-film transistors [23]. 

In the late 1980s, particular interest was also focused on the exploitation of this photocleavage reaction in chemical amplification concepts. Willson and co-workers synthetized poly(carbonate)s with *o*-nitrobenzyl pendant groups, which form free hydroxymethyl side groups upon UV exposure [24]. Chemically amplified depolymerization of the illuminated polymer was obtained during a post-baking step in the presence of a photo acid. A route towards positive chemical amplification was pursued by Wilkins et al., who described the synthesis of an *o*-NB ester of cholic acid [25]. The ester acted as photosensitive dissolution inhibitor for copolymers of methyl methacrylate and methyl acrylic acid. Deep UV exposure changed the solubility characteristics of the inhibitor since the cleavage products were highly soluble in alkaline solutions. In particular, positive tone patterns with a resolution of 0.5 µm were accomplished. 

Following the concept of an UV induced change in solubility, the application of *o*-NBE chemistry has also been expanded to patterned polymers used in biomedical applications. Doh and co-workers synthetized patternable acrylic copolymers with *o*-NBE pendant groups, which were applied in the fabrication of multicomponent protein and cell arrays [26]. Going a step beyond the fabrication of simple patterns, Li et al. realized reactive micropatterns by preparing new photodegradable polymers with *o*-NBE links in the main chain [27]. Since the *o*-NBE units were carrying additional functional groups such as epoxy, mercapto, or alkine moieties, the patterns could be modified by subsequent thiol–ene chemistry or copper catalyzed azide–alkyne cycloaddition reactions.

Inspired by this work, we employed *o*-NBE chemistry for the fabrication of positive tone photoresists by controlled degradation of thermally cured epoxy-based networks upon UV exposure [28,29]. The photosensitive links enabled the spatially controlled degradation of the covalent polymer networks upon UV exposure and a switch both in solubility properties and thermo-mechanical performance. By using a newly synthetized *o*-NBE derivative with terminal epoxy groups, photosensitive duromer networks were prepared by a thermal curing step in the presence of an anhydride hardener. The spatial control of the network degradation was successfully exploited to prepare positive tone micropatterns. 

**Figure 1:**
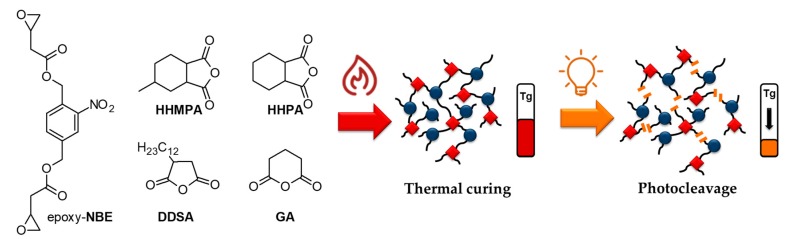
Epoxy-**NBE** as photosensitive epoxy-based monomer and cycloaliphatic dicarboxylic acid anhydrides used in the preparation of photopatternable thermosets.

Whilst the feasibility of the concept has already been shown in our previous work [28], the current work aims at the establishment of structure-property relationships between network composition and material properties (prior to and after photocleavage) to enhance the performance of the functional networks (Figure 1). In a comprehensive way, the anhydride hardener was varied to obtain networks with glass transition temperatures ranging between 16 and 72 °C. The results revealed that the differences in network mobility significantly affect the efficiency of the cleavage reaction, the formation of soluble species, and, thus, the contrast curve of respective positive tone photoresists.

## 2. Materials and Methods 

### 2.1. Materials and Chemicals

The anhydride hardeners and *N*,*N*- dimethylbenzylamine as accelerator were purchased from Sigma-Aldrich (St. Louis, MO, USA) and were used without further purification. (2-Nitro-1,4-phenylene) bis(methylene) bis(2-(oxiran- 2-yl)acetate) (epoxy-**NBE**) was synthesized as previously reported in ref. [28]. Caution: For preparative work, hazardous chemicals and solvents were employed. Reactions must be carried out in a fume hood, and protective clothes and goggles must be used.

### 2.2. Preparation and Thermal Curing of Photopatternable Epoxy-Based Thermosets

For sample preparation, epoxy-**NBE** was mixed with 1.0 epoxide equiv. of the respective anhydride hardener (Figure 1) and 0.1 wt% (related to the total reaction mixture) of *N*,*N*- dimethylbenzylamine. The composition of the resin formulations is summarized in Table 1. Regarding formulations containing either dodecenylsuccinic anhydride (DDSA) or hexahydro-4-methylphthalic anhydride (HHMPA), the components were stirred in a glass vial at room temperature for 30 min. In contrast, formulations with hexahydrophthalic anhydride (HHPA) or glutaric anhydride (GA) were heated to 50 °C in a water bath (to dissolve the anhydride in epoxy-**NBE**) and stirred for 30 min. Thermal curing of all formulations was carried out at 100 °C for 18 h yielding solid samples. 

### 2.3. Characterization of Curing and Cleavage Kinetics

To monitor the progress of the thermally induced ring-opening reaction between epoxy-**NBE** and the anhydride hardener, as well as the subsequent photo-induced cleavage of the *o*-NBE links, FT-IR spectroscopy with a Vertex 70 spectrometer (Bruker, Billerica, United States) was carried out. Sixteen scans were accumulated in transmission mode with a resolution of 4 cm^−1^. The areas of the IR absorption peaks were calculated with OPUS software (Version 7.0, Bruker, Billerica, MA, USA). For sample preparation, the resin formulations were drop-cast between a Si wafer and a CaF_2_ substrate and cured at 100 °C. The curing kinetics was monitored for 18 h and Fourier transform infrared (FT-IR) spectra were recorded at predefined times. 

Regarding the cleavage kinetics, the drop-cast films of the cured material were irradiated with a medium pressure Hg lamp (Omnicure S1000, Lumen Dynamics, ON, Canada) under nitrogen atmosphere. The cleavage reaction was followed upon prolonged UV exposure. To determine the light intensity (power density P; in mW cm^−2^) on the sample plane an integrated radiometer (Powerpuck II, EIT Instrument Markets, Leesburg, VA, USA) was used. The average power density was 114.4 mW cm^−2^ (between 250 and 470 nm).

### 2.4. Characterization of Network Properties

The glass transition temperature (*T*_g_) of cured samples was determined prior to and after UV exposure by differential scanning calorimetry (DSC) using a Mettler-Toledo DSC 821e (Columbus, OH, USA). For the analysis, the cured resins were heated from −20 to 120 °C with a heating rate of 20 °C min^−1^. The resulting *T*_g_ was characterized from the first heating run and was read as the midpoint in heat capacity. Regarding the sample preparation, 7–10 mg of the uncured resin formulation was placed into an aluminum crucible (Mettler-Toledo, 40 µL, Columbus, OH, USA) and cured for 18 h at 100 °C. Subsequent UV exposure was carried out as described in the previous section.

Nanoindentation experiments were performed to determine the surface hardness with the Ultra Nanoindentation Tester (Anton Paar, Graz, Austria) using a pyramidal-shaped Berkovich tip with an equivalent cone semi-angle q = 70.3 °. The contact force was 50 µN, and the maximum indentation force was set to 1000 µN. Both loading and unloading rate amounted to 6000 µN/min. A 30 s hold segment was applied at the maximum load to obtain reliable stress-strain curves. *H*_IT_ data were derived from the unloading-part of the load-displacement curve according to the “Oliver & Pharr Method” [30]. In this method, the indentation hardness (*H*_IT_) as a measure for surface hardness is equivalent to the mean pressure supported by the sample under maximum load. The distance between two measuring points was 150 µm, and nine indents were performed in total over an area of 300 × 300 µm to record the mechanical properties over a larger length scale and to get reliable results. 

### 2.5. Preparation and Characterization of Photopatterned Films

For sol-gel analysis, thin films were obtained by drop-casting the resin formulations between a Si waver and a polypropylene foil. After curing at 100 °C for 18 h, the polymer foil was removed, and the cured film was irradiated with a medium Hg lamp (Omnicure S1000, Lumen Dynamics, ON, Canada) under nitrogen for defined periods of time. To study the change of the gel fraction, FT-IR spectra (Vertex 70 spectrometer, Bruker, Billerica, MA, USA) of the non-exposed and UV exposed samples were taken after development in tetrahydrofuran for 10 min. Contrast curves were obtained by plotting the gel fractions versus the log function of the exposure dose.

Micropatterns were inscribed in the cured resin formulations by photolithography. Cured thin films were prepared as described in the previous paragraph. Photolithographic patterning was then performed with a quartz-chromium mask in contact mode with the Omnicure S1000 (Lumen Dynamics, ON, Canada) as light source. The exposure dose amounted to 22.3 J/cm^2^ for cured epoxy-**NBE**/HHMPA, epoxy-**NBE**/HHPA and epoxy-**NBE**/DDSA systems. In terms of epoxy-**NBE**/GA networks an exposure dose of 8.5 J/cm^2^ was applied. The polymer films were developed by dipping the samples for few seconds in tetrahydrofuran.

Surface topography measurements were conducted with the 3D optical surface metrology system Leica DCM8 (Leica Microsystems, Wetzlar, Germany). The images were obtained using a Mirau 20× objective and the ePSI (extended Phase Shift Interferometry) mode, with blue light. This allows for a theoretical optical resolution of 0.35 µm and a vertical resolution better than 1 nm.

## 3. Results and Discussion

### 3.1. Thermal Curing of Photopatternable Epoxy-Based Thermosets

**Figure 2:**
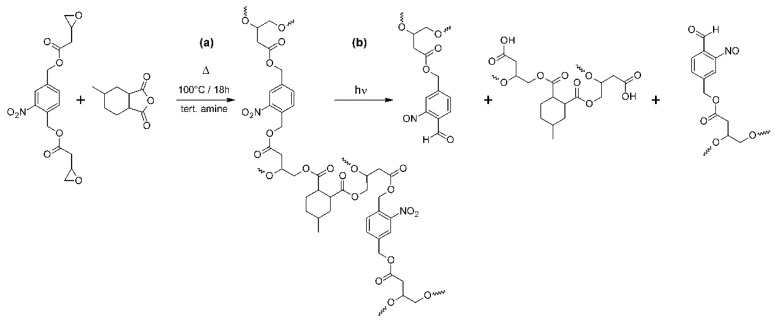
(**a**) Thermal curing and (**b**) photocleavage of epoxy-based thermosets with photosensitive *o*-NBE links.

Photopatternable thermosets were prepared by thermal curing of the epoxy-terminated *o*-NBE derivative (epoxy-**NBE**) with selected cycloaliphatic anhydrides (Figure 2a). Moreover, a tertiary amine was added as an accelerator to reduce both time and temperature of the cure reaction. The mechanism of the accelerated curing follows a ring opening reaction of the anhydride by the amine under the formation of betaines [6]. The internal salts provide carboxylate ions, which initiate the cure reaction by forming alkoxide esters with the epoxy groups. Once formed, the alkoxide esters react with further anhydride groups yielding carboxylate anion functional esters. Those are able to open epoxy moieties leading to a perpetuation of the ring-opening reaction.

**Figure 3:**
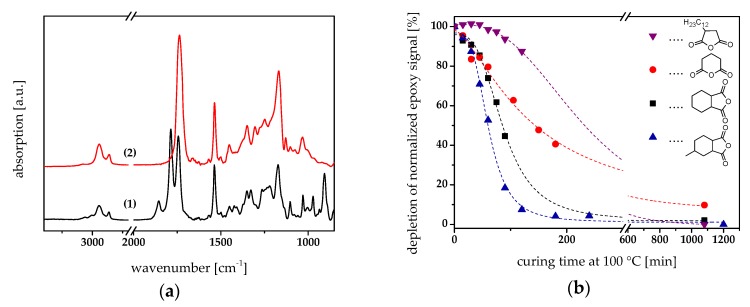
(**a**) Fourier transform infrared (FT-IR) spectra of epoxy-**NBE**/HHPA (1) prior to and (2) after thermal curing at 100 °C. (**b**) Influence of the anhydride structure on the cure kinetics of photopatternable epoxy-based thermosets by following the normalized depletion of the peak area between 910 and 928 cm^−1^. Thermal curing was performed at 100 °C. The lines are a guide for the eye.

For determination of the cure kinetics, the resin formulations were drop-cast between a CaF_2_ disc and a Si wafer. Figure 3a displays the FT-IR spectra of epoxy-**NBE**/HHPA prior to and after thermal curing at 100 °C. In the cured state, the two carbonyl peaks (1860 and 1778 cm^−1^) related to the anhydride group disappeared whilst a new band arose at 1740 cm^−1^, which can be assigned to the carbonyl unit of the formed ester linkages. In addition, the epoxy peak at 910 cm^−1^ disappeared confirming the successful network formation by the nucleophilic ring-opening reaction. Identical observations were made for the other epoxy/anhydride formulations under investigation (Figure S1a–c in Supplementary Materials). 

To study the influence of the anhydride structure on the cure kinetics, the depletion of the epoxy peak at 910 cm^−1^ was followed upon curing at 100 °C (Figure 3b). Since this epoxy band partly overlaps with an absorption band of the anhydride group at 920 cm^−1^, the depletion of both signals was monitored. The results reveal that the curing rate decreases in the order of HHMPA>HHPA>GA>DDSA. Whilst for MHHPA nearly full conversion was observed after a curing time of 180 min, prolonged curing (> 1000 min) is required, both for GA and DDSA, to reach a conversion above 90%. The long alky chain in its structure may explain the low reactivity of DDSA. On the one hand, it lowers the acidity of the anhydride groups and on the other hand, it might hamper the reaction due to steric effects. A comparable behavior was found for nonyl succinic anhydride in the non-catalyzed ring-opening of epoxy resins [31].

In contrast, the lower reactivity of GA compared to HHMPA or HHPA might be related to the higher stability (lower ring strain) of the six-membered ring system. Previous studies on the ring-opening copolymerization of epoxides and anhydrides showed that a decrease of the ring strain (of either epoxides or anhydrides) corresponds to a lower reactivity of the monomers and results in lower molecular weight products [32,33].

Along with cure kinetics, the structure of the anhydride affects thermo-mechanical properties and surface hardness, as shown in Table 2. From DSC measurements, it is obtained, that more rigid anhydrides (HHMPA and HHPA) yield networks with glass transition temperatures (*T*_g_) of 72 and 46 °C, respectively. In contrast, epoxy-**NBE**/GA networks comprise a *T*_g_ below room temperature, which is attributed to the flexible nature of the anhydride in its opened state. In contrast, curing with DDSA gives networks with a *T*_g_, which is only slightly lower (44 °C) than the epoxy-**NBE**/HHPA system. DDSA cured epoxy thermosets with a *T*_g_ in the range of 40 °C were also reported by Webster and Pan, who established structure-property relationships of epoxy-based resins from renewable resources [34].

The indentation hardness (*H*_IT_) of the cured resins under investigation correlates well with the respective *T*_g_ values and increases in the order of GA>DDSA>HHPA>HHMPA. Depending on the structure of the anhydride, the surface hardness varied over three orders of magnitude: from the soft epoxy-**NBE**/GA system with a surface hardness of 8.6 ± 0.8 MPa to the rigid epoxy-**NBE**/HHMPA network with 213.5 ± 19.8 MPa.

### 3.2. Photocleavage of Photopatternable Epoxy-Based Thermosets

The photocleavage of the epoxy-based networks was subsequently monitored in cured films, which were drop-cast between a CaF_2_ disc and a Si wafer. The samples were irradiated with UV-light at wavelengths below 400 nm to induce the cleavage reaction of the *o*-NBE links. The ester photolysis typically yields nitroso compounds and carboxylic acids as primary cleavage products (Figure 2b) [35]. In the present study, the cleavage is evidenced by the depletion of the two characteristic NO_2_ signals at 1537 cm^−1^ and 1348 cm^−1^ (conversion of nitro groups to nitroso moieties). The related spectra are provided in Figure S2a–d in Supplementary Materials. In addition, a distinctive broadening of the C=O absorption band (1646–1830 cm^−1^) is observed, which is associated with the formation of carboxylic acids and other carbonyl-based cleavage products. 

**Figure 4:**
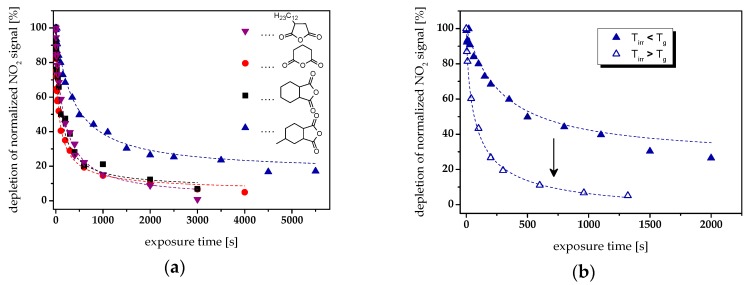
(**a**) Cleavage kinetics of photopatternable epoxy-based thermosets versus anhydride structure. The normalized depletion of the nitro groups (at 1537 cm^−1^) is followed upon prolonged UV irradiation (74.5 mW cm^−2^). The lines are a guide for the eye. (**b**) Normalized depletion of the nitro groups (at 1537 cm^−1^) in epoxy-**NBE**/HHMPA networks as a function of the reaction temperature: (full triangles) no additional heating of the sample during UV exposure (*T*_irr_ < *T*_g_) and (open triangles) simultaneous heating of the sample to 80 °C (*T*_irr_ > *T*_g_). The lines are a guide for the eye.

The cleavage kinetics was determined as a function of the employed anhydride by following the depletion of the NO_2_ signal at 1537 cm^−1^ upon prolonged UV irradiation (Figure 4a). A rapid decrease of the nitro absorption band was observed in the low-T_g_ network epoxy-**NBE**/GA. This is in good agreement with previous studies, in which we have reported that the photocleavage kinetics of *o*-NBE links is influenced by the mobility of the chromophore [36]. However, it is interesting to note that epoxy-**NBE**/HHPA and epoxy-**NBE**/DDSA networks, whose *T*_g_ is above room temperature (46 and 44 °C, respectively), comprise a cleavage rate that is equal to the epoxy-**NBE**/GA system. This might be explained by the temperature of the sample’s surface, which rises to 38–40 °C during UV exposure. Consequently, the UV exposure is carried out either above or close to the *T*_g_, which ensures a high mobility of the networks resulting in comparable cleavage kinetics. 

In contrast, the high-T_g_ network (72 °C) epoxy-**NBE**/HHMPA is characterized by a significantly lower cleavage rate due to the lower mobility of the chromophore under irradiation conditions. This can be easily overcome by simultaneously heating the sample to 80 °C (*T*_irr_ > *T*_g_) during UV exposure. As shown in Figure 4b, the higher temperature significantly accelerates the cleavage rate and leads to a higher conversion of the nitro groups, confirming the important role of network mobility on the cleavage kinetics of *o*-NBE based networks.

The photo-induced degradation of the networks is reflected by the decrease of the *T*_g_ as well as the surface hardness (Table 2). The optically triggered change in material properties is more pronounced in networks containing rigid anhydrides such as HHMPA or HHPA. In particular, for epoxy-**NBE**/HHMPA systems, the *T*_g_ decreases from 72 to 48 °C, whilst in terms of epoxy-**NBE**/GA networks, the change in the *T*_g_ does not exceed 4 °C. With respect to the surface hardness, a similar trend was observed. 

In further experiments, the influence of simultaneous heating was studied by measuring the surface hardness of the high-T_g_ network epoxy-**NBE**/HHMPA as a function of the reaction temperature. Without additional heating, the surface hardness is nearly halved by the UV-induced network degradation and the *H*_IT_ value decreases from 213.5 ± 19.8 to 110.9 ± 10.3 MPa. By simultaneously heating the sample to either 50 or 70 °C, the *H*_IT_ values further decreased to 40.6 ± 3.8 and 29.1 ± 2.7 MPa, respectively. From the results, it can be concluded that the cleavage of *o*-NBE linkages facilitates network degradation, whose efficiency can be significantly increased by rising reaction temperature and, thus, mobility of the thermosets. 

### 3.3. Photopatterning Studies

For a successful positive tone patterning of the epoxy-based networks under investigation, the photo-induced formation of soluble species is crucial. Thus, sol-gel analysis was performed on thin cured resins, which were UV irradiated for selected exposure doses and developed in tetrahydrofuran. The insoluble fraction was then determined by quantitative FT-IR spectroscopy. Figure 4a–d show the gel fraction of the four thermosets versus exposure time. 

**Figure 5:**
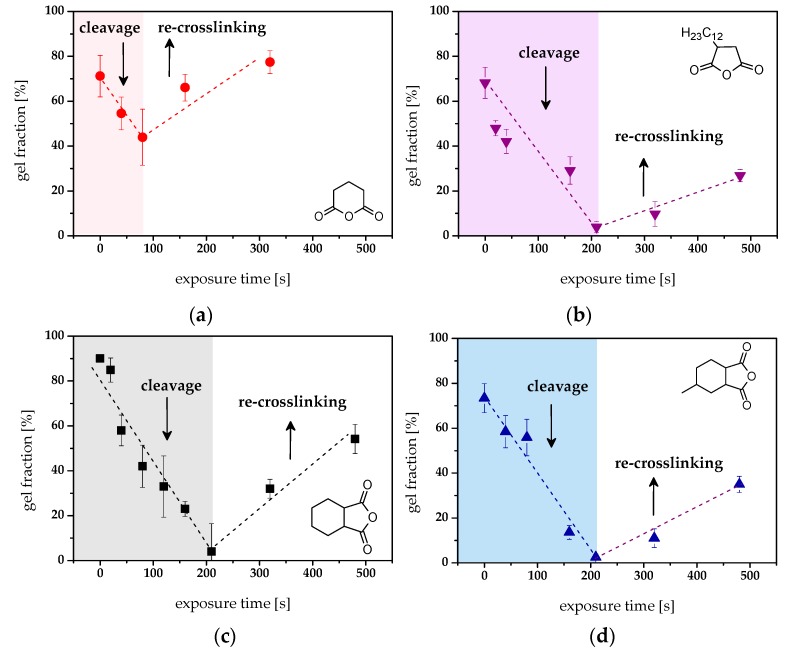
Gel fraction of (**a**) epoxy-**NBE**/GA, (**b**) epoxy-**NBE**/DDSA, (**c**) epoxy-**NBE**/HHPA and (**d**) epoxy-**NBE**/HHMPA versus exposure time (75 mW/cm^2^) as derived from quantitative FT-IR measurements.

Whilst epoxy-**NBE**/GA, epoxy-**NBE**/DDSA, and epoxy-**NBE**/HHPA networks were characterized by comparable cleavage rates and cleavage yields (Figure 3a), their ability to form soluble cleavage products differs significantly. For the low-T_g_ network epoxy-**NBE**/GA, a gradual decrease of the gel fraction is observed with rising UV irradiation until a minimum of 42% is reached (Figure 5a). A further increase in the exposure time leads to an increase in the gel fraction, which is explained by a reformation of covalent links. Previous work showed that secondary cleavage products are readily formed in highly mobile polymer networks (e.g., formation of azobenzene moieties) leading to a re-crosslinking of the networks [29,36]. In contrast, the epoxy-**NBE**/DDSA network with a *T*_g_ above room temperature becomes fully soluble at an exposure time of 200 s (Figure 5b). At prolonged UV exposure (> 200 s), a re-crosslinking of the cleaved polymer chains was again observed. Networks with rigid anhydrides showed the same behavior (Figure 5c,d). The results confirm that mobile *o*-NBE networks are more prone to side reactions compared to rigid ones. This correlates well with the UV-induced changes in surface hardness and *T*_g_, which are less pronounced in flexible networks. Similar behavior was also observed in recent studies on photocleavable thiol-ene, thiol-yne, and polydimethyl siloxane networks, which have a *T*_g_ below room temperature and are highly susceptible to re-crosslinking due to secondary photoreactions [36,37,38,39].

**Figure 6:**
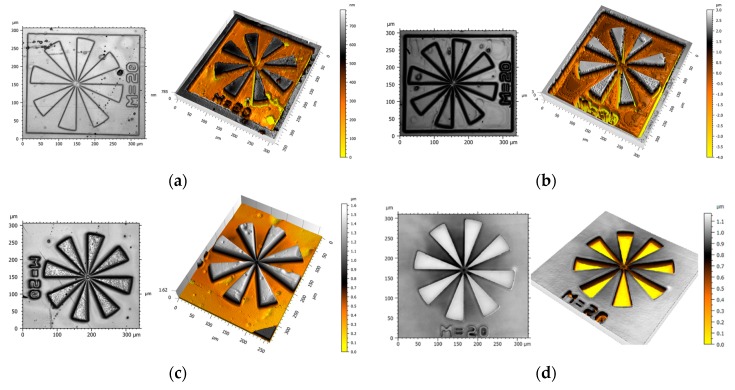
Micrographs and surface profiles of photopatterned (**a**) epoxy-**NBE**/GA, (**b**) epoxy-**NBE**/DDSA, (**c**) epoxy-**NBE**/HHPA, and (**d**) epoxy-**NBE**/HHMPA systems as obtained from interference microscopy.

**Figure 7:**
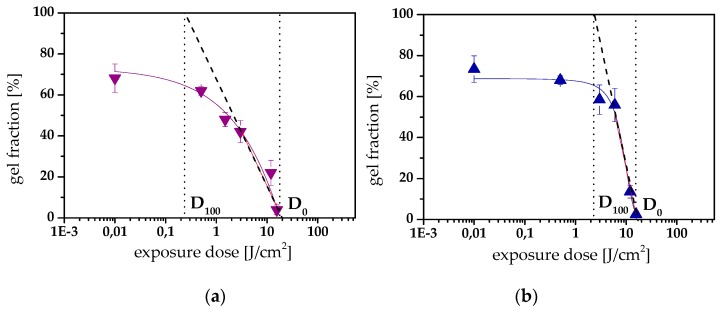
Contrast curves of (**a**) epoxy-**NBE**/DDSA and (**b**) epoxy-**NBE**/HHMPA networks and calculation of the contrast (*γ*) from the slope of the linear area (dashed line) of the curve by extrapolating it to a gel content of 0% (D_0_) and 100% (D_100_), respectively.

Although epoxy-**NBE**/GA systems suffer from incomplete network degradation, the formation of soluble products is reasonably efficient for inscribing positive tone relief structures in the thermosets by photolithography (Figure 6a). For the other three resin systems, which are becoming fully soluble upon UV exposure, characteristic photoresist properties, such as contrast and resolution, were determined (Table 3). The contrast is a measure of the solubility change of the resist upon UV exposure, and contrast curves were determined by plotting the gel fraction (which corresponds to the thickness of the resist layer after the development step) as a log function of exposure dose (Figure 7a,b). The contrast (*γ*) was calculated according to the following equation:

(1) $$\gamma =\;\frac{1}{\log\left(\frac{D_{0}}{D_{100}}\right)}\;,$$

in which *D*_0_ and *D*_100_ correspond to the exposure dose obtained by taking the slope of the linear portion of the contrast curve and extrapolating it to a gel content of 0% and 100%, respectively [40]. From the contrast curves, it can be obtained that the exposure dose at which complete solubility is reached (*D*_100_) is in a similar range for all three networks under investigation. However, the *D*_0_ value of epoxy-**NBE**/HHMPA systems was significantly lower than in epoxy-**NBE**/DDSA and epoxy-**NBE**/HHPA networks, which resulted in a higher contrast (1.17 versus 0.53). 

Topography images of the positive tone patterns inscribed in epoxy-**NBE**/DDSA, epoxy-**NBE**/HHPA and epoxy-**NBE**/HHMPA systems are shown in Figure 6b–d. Whilst the exposed areas facilitated a rapid development, no significant swelling was observed in the crosslinked areas, which have not been illuminated. 

In a subsequent step, the resolution was determined by pursuing a classical approach with patterns comprising lines and spaces with varying widths and by investigating the polymer patterns after the development. For epoxy-**NBE**/HHMPA networks, separate lines and spaces of the positive tone patterns were observed until a pattern width of 8 µm was reached. In contrast, epoxy-**NBE**/HHPA and epoxy-**NBE**/DDSA networks are characterized by a lower resolution (> 10 µm). This correlates well with the lower contrast observed in these two thermosets. It should be further noted that the contrast is lower compared to resists used in the microelectronic industry, which typically have a contrast in the range of 2–3 [40]. However, the photo-induced conversion of an insoluble thermoset to soluble cleavage products paves the way toward a versatile and convenient micropatterning of epoxy-based duromers. 

## 4. Conclusions

Structure–property relationships of photopatternable epoxy-based thermosets were established clearly, showing the important role of network structure and network mobility on the cleavage kinetics, material properties, and related patterning performance of the photosensitive networks. Network mobility of the thermosets was conveniently adjusted by either varying the anhydride hardener or by increasing the reaction temperature during UV irradiation. Along with cure kinetics, the anhydride hardener affects material properties such as *T*_g_ and surface hardness. In particular, low-T_g_ networks (epoxy-**NBE**/GA) with a *T*_g_ of 16 °C as well as high-T_g_ networks (epoxy-**NBE**/HHMPA) with a *T*_g_ of 72 °C were obtained. The network mobility strongly influences the photolysis of the *o*-NBE linkages. High-T_g_ networks are characterized by a lower cleavage rate, which can be easily overcome by heating the networks above their *T*_g_ during UV irradiation. However, sol-gel analysis reveals that flexible networks are prone to secondary photoreactions. The side reactions lead to a re-crosslinking of the network and an incomplete formation of soluble species. In contrast, networks with a *T*_g_ above room temperature become fully soluble due to the photolysis of the *o*-NBE chromophores. Thus, although high-T_g_ networks suffer from lower cleavage rates, they benefit from a better patterning performance. In particular, epoxy-**NBE**/HHMPA systems facilitate a resolution of 8 µm and a contrast of 1.17 without any optimization.

## Figures and Tables

**Table 1:** Composition of photopatternable epoxy-based resin formulations.

Resin Formulation	Type of Anhydride	Molar Ratio ^1^	Accelerator [wt%]
epoxy-**NBE**/HHMPA	HHMPA	1	0.1
epoxy-**NBE**/HHPA	HHPA	1	0.1
epoxy-**NBE**/DDSA	DDSA	1	0.1
epoxy-**NBE**/GA	GA	1	0.1

^1^ Molar ratio of anhydride crosslinker to epoxy-**NBE** in resin formulation.

**Table 2:** Glass transition temperature (*T*_g_) and indentation hardness (*H*_IT_) of cured photopatternable epoxy-based thermosets prior to and after UV exposure (74.5 mW cm^−2^).

Resin Formulation	*T*_g_ [°C]	*H*_IT_ [MPa]	*T*_g_ [°C]	*H*_IT_ [MPa]
	Thermally Cured		Photocleaved	
epoxy-**NBE**/HHMPA	72	213.5 ± 19.8	48	110.9 ± 10.3
epoxy-**NBE**/HHPA	46	148.6 ± 13.8	32	83.0 ± 6.3
epoxy-**NBE**/DDSA	44	51.0 ± 4.7	30	41.3 ± 3.8
epoxy-**NBE**/GA	16	8.6 ± 0.8	12	1.3 ± 0.1

**Table 3:** Characteristics of positive tone photoresists based on photocleavable epoxy-based thermosets.

Resin Formulation	*D*_0_ [J/cm^2^]	*D*_100_ [J/cm^2^]	*γ*	Resolution [µm]
epoxy-**NBE**/HHMPA	16.3	2.27	1.17	8
epoxy-**NBE**/HHPA	19.5	0.26	0.53	>10
epoxy-**NBE**/DDSA	19.7	0.25	0.53	>10

## Supplementary Materials

The following are available online at https://www.mdpi.com/1996-1944/12/15/2350/s1, Figure S1: FT-IR spectra of (**a**) epoxy-**NBE**/GA, (**b**) epoxy-**NBE**/DDSA, and (**c**) epoxy-**NBE**/HHMPA prior to and after thermal curing at 100 °C, Figure S2: FT-IR spectra of (**a**) epoxy-**NBE**/GA, (**b**) epoxy-**NBE**/DDSA, and (**c**) epoxy-**NBE**/HHMPA prior to and after photocleavage.
