# Supplementary material for: Photopatternable Epoxy-Based Thermosets
Source: Materials (Basel). 2019 Jul 24;12(15):2350. doi: 10.3390/ma12152350 (PMC6695657; doi:10.3390/ma12152350)
Supplement: Supplementary file 1 [file materials-12-02350-s001.pdf]

Supplementary Materials

Photopatternable Epoxy-Based Thermosets

Michael Giebler [1], Simone Radl [1], Thomas Ules [1], Thomas Griesser [2], and Sandra Schlögl [1,*]

[1] Polymer Competence Center Leoben GmbH, Roseggerstrasse 12, A-8700 Leoben, Austria; Michael.Giebler@pccl.at (M.G.); Simone.Radl@pccl.at (S.R.); Thomas.Ules@pccl.at (T.U.)

[2] Institute of Chemistry of Polymeric Materials, Montanuniversitaet Leoben, Otto Glöckel-Strasse 2, A-8700 Leoben, Austria; Thomas.Griesser@unileoben.ac.at

* Correspondence: Sandra.Schloegl@pccl.at; Tel.: +43-3842-402-2354

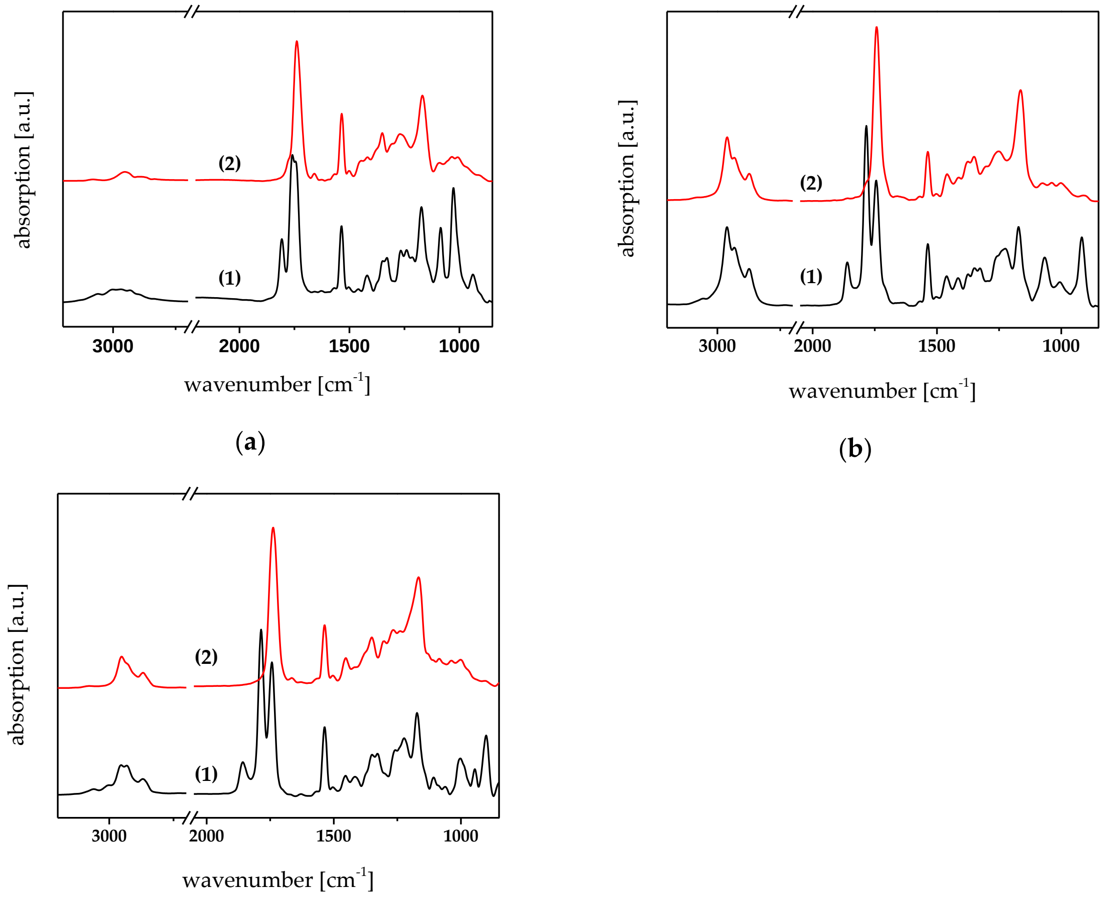

(c)

Figure S1. FT-IR spectra of (**a**) epoxy-**NBE**/GA, (**b**) epoxy-**NBE**/DDSA and (**c**) epoxy-**NBE**/HHMPA (1) prior to and (2) after thermal curing at 100 °C.

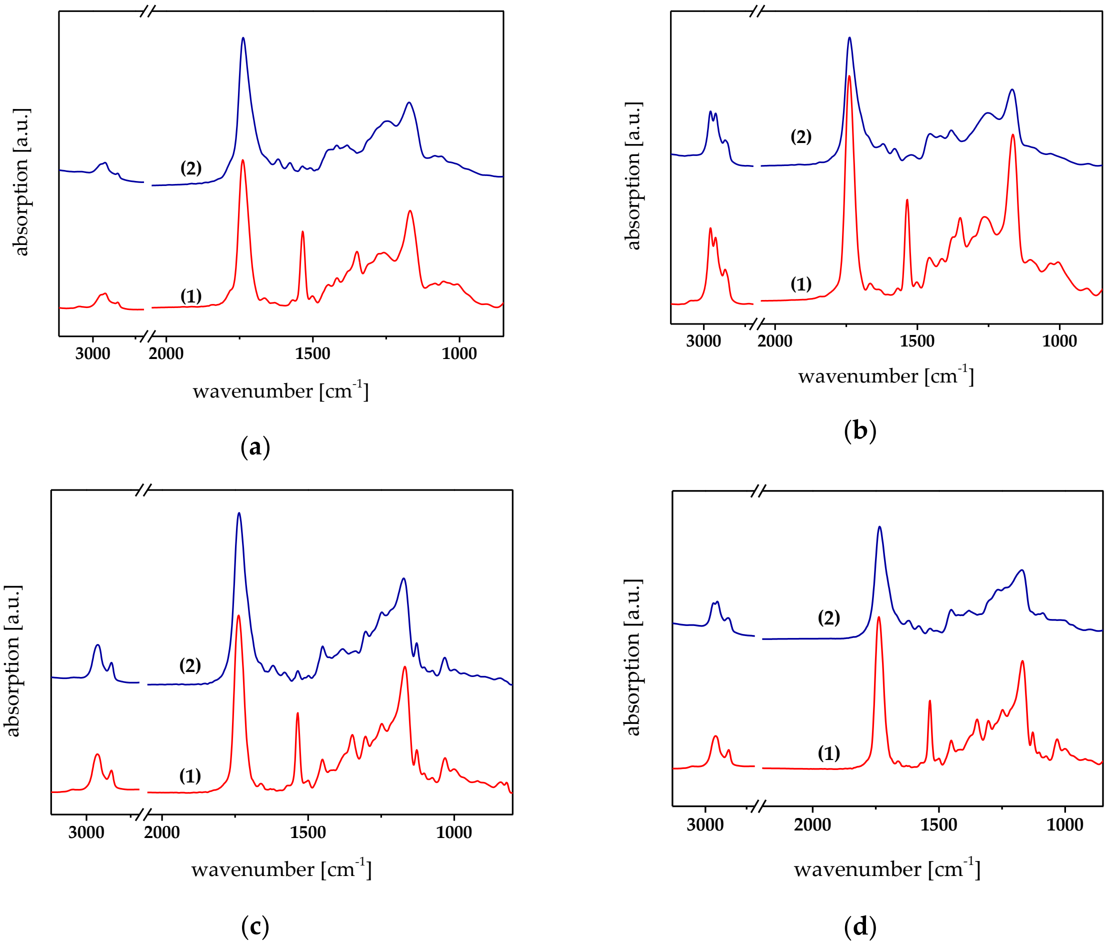

Figure S2. FT-IR spectra of (**a**) epoxy-**NBE**/GA, (**b**) epoxy-**NBE**/DDSA and (**c**) epoxy-**NBE**/HHMPA (1) prior to and (2) after photocleavage (74.5 mW cm^{-2}).
